# Neuroblastoma, Body Mass Index, and Survival: A Retrospective Analysis

**DOI:** 10.1097/MD.0000000000000713

**Published:** 2015-04-10

**Authors:** Annabel G. Small, Le M. Thwe, Jennifer A. Byrne, Loretta Lau, Albert Chan, Maria E. Craig, Chris T. Cowell, Sarah P. Garnett

**Affiliations:** From the School of Medicine (AGS, MEC), University of New South Wales; Children's Cancer Research Unit (LMT, JAB, LL), Sydney Children's Hospitals Network (Westmead); Discipline of Paediatrics and Child Health (LMT, JAB, LL, CTC, SPG), Sydney Children's Hospitals Network (Westmead) Clinical School, University of Sydney; Kids Research Institute (LMT, JAB, LL, CTC, SPG); Institute of Endocrinology and Diabetes (AC, MEC, CTC, SPG), Sydney Children's Hospitals Network (Westmead); and School of Women's and Children's Health (MEC), University of New South Wales, Sydney, New South Wales, Australia.

## Abstract

Extremes of body mass index (BMI) at diagnosis of childhood cancers have been associated with poorer prognosis.

The aims of this retrospective review were to examine the growth and BMI status of children diagnosed with neuroblastoma (NB) and determine if BMI status at diagnosis affected survival.

Between 1985 and 2005, 154 children were diagnosed with NB at Sydney Children's Hospitals Network (Westmead), Australia, of which 129 had both length/height and weight recorded in the medical records at diagnosis. BMI was calculated and children were classified as underweight (BMI <15th percentile), normal weight, and overweight (BMI >85th percentile). Disease stage was classified according to the International NB Staging System.

At diagnosis, 24.0% of the children were classified as underweight and 11.6% were overweight. Six months after diagnosis all children except those with stage 4s disease had a decrease in BMI z-score; difference in estimated marginal mean −0.73, *P* < .001. After 12 months an increase in BMI z-score was observed and by 2 years BMI z-score was significantly higher than BMI z-score at baseline; difference in estimated marginal mean 0.81, *P* = .007. At the last follow-up (median 5.6 years [range 3–7] after diagnosis) the proportion of children who were classified as underweight decreased to 8.7% and the proportion of children who were classified as overweight increased to 27.5%. The overall survival rate was 61.2%; however, BMI status did not predict survival. In multivariable Cox regression modeling, stage at diagnosis was the only predictor of survival; children diagnosed with stage 4 were less likely to survive (hazard ratio [HR] [95%CI]: 7.02 [1.7–29.0], *P* = .007).

Almost a quarter of children with NB were underweight at diagnosis. However, we did not demonstrate a prognostic association between BMI status and survival. The high proportion of children who were classified as overweight at follow-up indicates a need for nutritional interventions to prevent potential late effects.

## INTRODUCTION

Neuroblastoma (NB) is one of the most common solid tumors in children.^1^ In Australia, NB survival rates have increased from 52.9% in 1994 to 68.4% in 2004^2^ due to advances in multimodal therapy and increased understanding of the disease's genetic basis.^3^ While children with NB have one of the lowest 5-year survival rates of all childhood cancers in Australia, survival rates vary greatly according to disease stage, from 95.6% for stage 1 disease to 49.8% for stage 4.^2^ This creates a dual need to address the long-term effects of the disease in survivors, while continuing to identify modifiable risk factors to improve prognosis in these children.^4^

In adults, obesity at diagnosis of cancer has been repeatedly identified as a negative prognostic indicator of morbidity and mortality.^5,6^ Obesity is also associated with reduced survival and increased treatment-related complications in children diagnosed with acute lymphoblastic leukemia,^7^ acute myeloid leukemia,^8^ and osteosarcoma.^9^ Several studies have also identified obesity, measured by BMI, as a treatment-related outcome for some childhood cancers, particularly acute lymphoblastic leukemia.^10^ Due to the substantial impact obesity can have on a child's physical and mental well-being, the issue of treatment-related obesity may be a concern in children with NB.^11,12^

Children with NB are typically characterized as being underweight at diagnosis,^13^ having impaired growth following some modes of treatment,^11^ and at risk of a variety of late effects, including prediabetes, diabetes, and hypothyroidism.^11^ However, there is a paucity of data on the effect of BMI at diagnosis on survival of these children and their weight patterns during treatment and at long-term follow-up have not been previously explored. The objectives of this study were to examine the growth and BMI status of children diagnosed with NB and determine if BMI status at diagnosis of NB affects survival.

## METHODS

This study was a retrospective review of children (n = 154) diagnosed with NB between January 1985 and December 2005 at the Sydney Children's Hospitals Network (SCHN) (Westmead), Australia. Eligible children were identified through the Oncology Database at the SCHN (Westmead). Diagnoses of eligible children were reviewed using medical records and histopathology reports and confirmed according to the International Neuroblastoma Pathology Classification.^14^ Data on disease parameters were collected from medical records and included age, disease stage at diagnosis, date of diagnosis, date of last follow-up, and disease status at last follow-up. Disease stage was classified according to the International Neuroblastoma Staging System.^15^ Survival time was defined as the number of years from diagnosis to death from any cause, or for children who were alive at last follow-up, as the number of years from diagnosis to last follow-up. Median follow-up time was 5.6 years [range 3–7]. The study was approved by the SCHN Human Research Ethics Committee (LNR-2011–10–11). All patient data were analyzed and reported in a deidentified fashion. As the Oncology database includes identifying patient information, it is not a public resource. Deidentified data from this cohort could be made available for collaborative research, subject to further Human Research Ethics Committee approval. In this case, interested researchers should contact the corresponding author (S. P. G.).

### Anthropometry

Length/height and weight measurements at diagnosis, 3 months, 6 months, 1 year, 2 years, and last follow-up after diagnosis were retrieved from medical records. Measurements were collected as close to these times as possible, provided they fell within a specified range, Table 1. When more than 1 measurement was available within the range for a specific time point, the measurement closest to the specified time was used. BMI was calculated as weight (kg) divided by length/height (m).^2^ Length/height, weight, and BMI z-scores were derived from age and gender-specific reference values, using the British 1990 Growth reference data and the LMS Growth Program.^16^ Underweight was defined as <15th percentile (BMI z-score <−1.040), normal weight as 15th to 85th percentile (−1.036 <BMI z-score <1.030) and overweight as >85th percentile (BMI z-score >1.036).^17^ For survival analysis, children were also classified according to weight z-score at diagnosis. Low weight was defined as <15th percentile (weight z-score <−1.040), average weight as 15th to 85th percentile (−1.036 <weight z-score <1.030), and heavy weight as >85th percentile (weight z-score >1.036).^17^

**TABLE 1:**
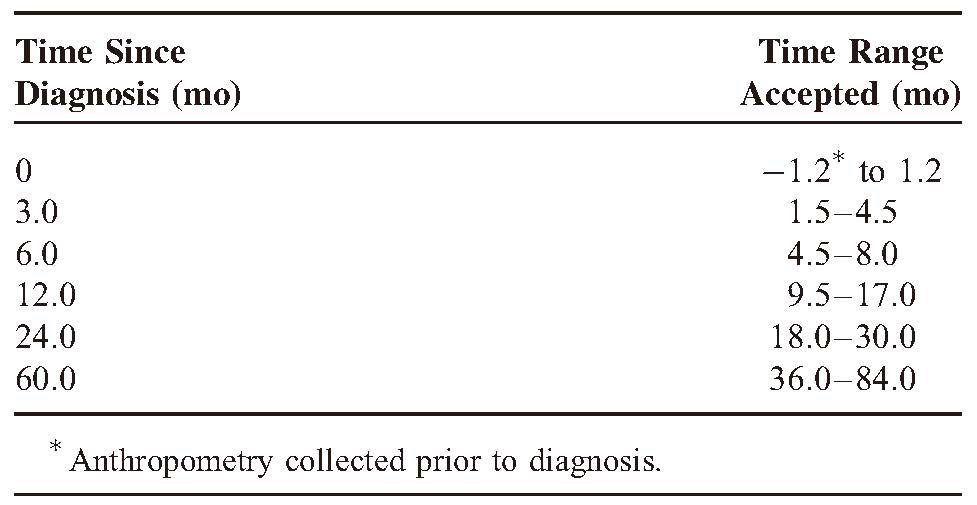
Time Ranges Used to Group Anthropometric Data of Children Diagnosed With Neuroblastoma

### Statistical Analysis

Data were analyzed using the Statistical Package for Social Sciences, version 20.0 (SPSS, Chicago, IL). Categorical data are summarized as frequencies and proportions and differences between groups were examined using the χ^2^ test. Continuous data are summarized as medians (25th, 75th confidence interval [CI]) and differences between groups were compared using Kruskal–Wallis test for multiple independent samples and Mann–Whitney U test for 2 independent samples. To optimize the use of all available anthropometric data, linear mixed models with an unstructured covariance structure were used to test for the effects of gender, age, disease stage at diagnosis, and time after diagnosis, as well as a stage × time interaction on height, weight, and BMI z-scores. For the mixed modeling analysis, NB staging was categorized as either stage 1 or 2, stage 3 or 4 and 4s. Gender and age were not statistically or clinically significant in the models and were not included in the final models. The least significant difference method was used for post hoc comparisons. The assumptions of modeling were tested and met. Survival probabilities for the underweight, normal weight, and overweight children were calculated using the Kaplan–Meier method. Survival differences between these groups were compared using the log-rank test. Cox regression models were used to calculate HR, estimating the effect of age, disease stage, and BMI and weight z-score at diagnosis on overall survival. Statistical significance was set at *P* < 0.05.

## RESULTS

Between January 1985 and December 2005, 154 children (boys, n = 89; 58%) were diagnosed with NB, of which 129 had length/height and weight measurements recorded at diagnosis. The median age at diagnosis was 1.2 years (range 0–10.6 years) and 31 children (24.0%) were classified as underweight and 15 (11.6%) as overweight. There was no significant difference in gender, age, or disease stage at diagnosis between children who were classified as underweight, normal weight, or overweight, Table 2.

**TABLE 2:**
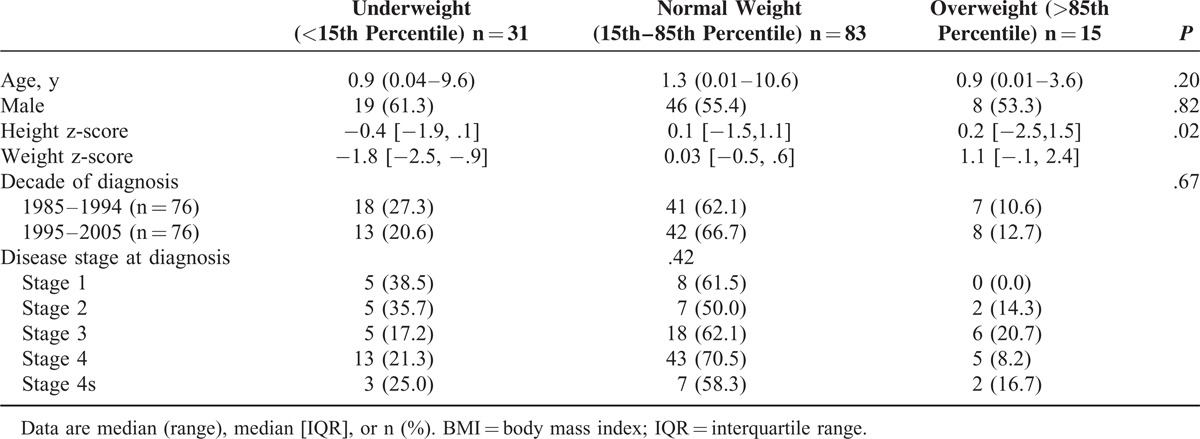
Characteristics of Children Diagnosed With Neuroblastoma Stratified by BMI Status at Diagnosis (n = 129)

### Height, Weight and BMI Changes From Diagnosis to Last Follow-up, Figure 1

**FIGURE 1:**
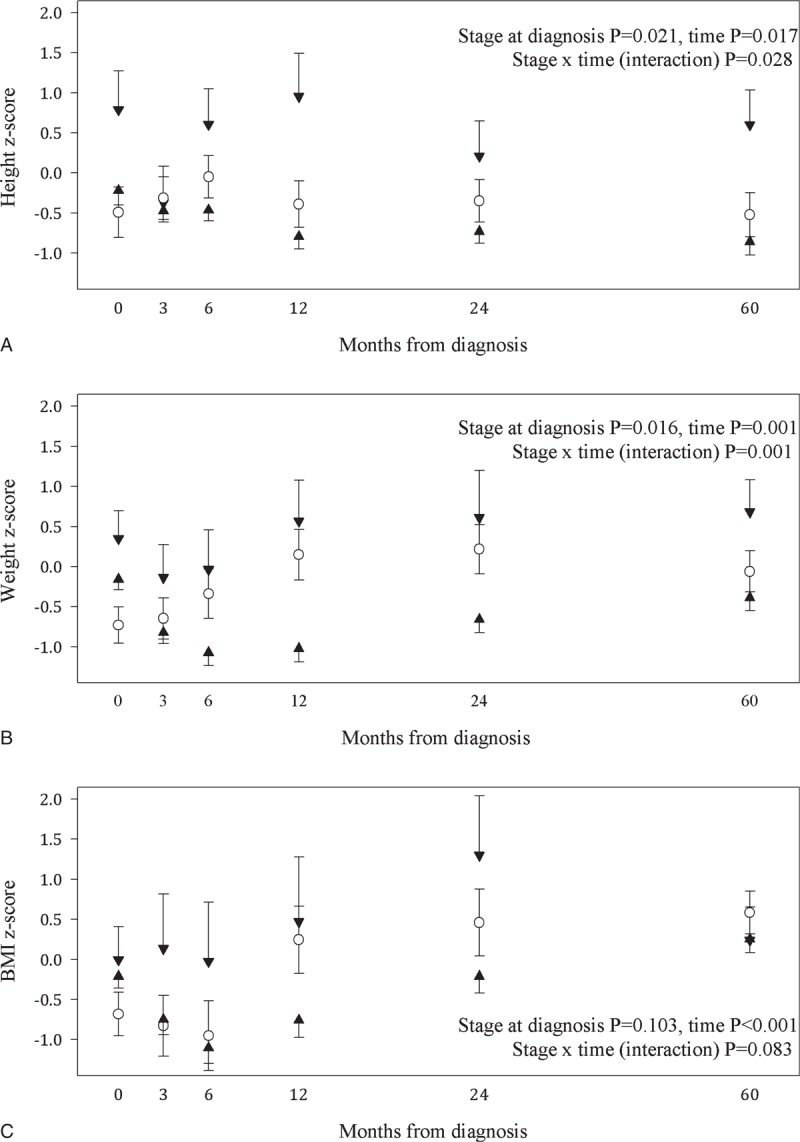
Height (A), weight (B), and BMI (C) z-scores by stage of neuroblastoma at diagnosis. Estimated marginal means and standard errors are presented from linear mixed models for NB stage 1 and 2: n = 27; white circle. Bars represent positive and negative standard errors NB stage 3 and 4: n = 90; black up triangle. Bars represent negative standard errors. NB stage 4s n = 12; black triangle down. Bars represent positive standard errors.

Disease stage at diagnosis (*P* = .021) and time since diagnosis (*P* = .017) predicted height z-scores, Figure 1A. Children with stage 4s had a higher height z-score at all-time points compared to those diagnosed with stage 1 to 4 NB. Children with stage 3 or 4 NB demonstrated a slowing of linear growth (z-score decreased by 0.44) in the first 6 months of treatment and the decrease in relative height was not regained at last follow-up; EMM [95% CI] at diagnosis −0.02 [−0.38 to 0.33] compared to −0.86 [−1.19 to −0.53] at last follow-up.

Disease stage at diagnosis (*P* = .016) and time since diagnosis (*P* = .001) were also predictors of weight z-scores, Figure 1B. Children with stage 3 or 4 and 4s NB lost weight in the first 6 months of treatment which was followed by weight gain. However, they were lighter at last follow-up (weight z-score EMM -0.39 [−0.72 to −0.06]) compared to diagnosis (−0.16 [−0.42 to 0.11]).

Time from diagnosis was a predictor of BMI z-score (*P* < .001), but disease stage was not a significant predictor (*P* = .103), Figure 1C. Children diagnosed with all stages of NB, except for stage 4s, had a decrease in BMI z-score over the first 6 months; difference in EMM 0.73, *P* < .001. Overall, an increase in BMI z-score was evident at 12 months and by 2 years BMI z-score was significantly higher (*P* = .007) compared to BMI z-score at diagnosis and remained higher at last follow-up (*P* = .002). During this time, the proportion of children who were classified as underweight decreased from 24.0% to 8.7% and overweight increased from 11.6% to 27.5%.

### Survival Analysis

**FIGURE 2:**
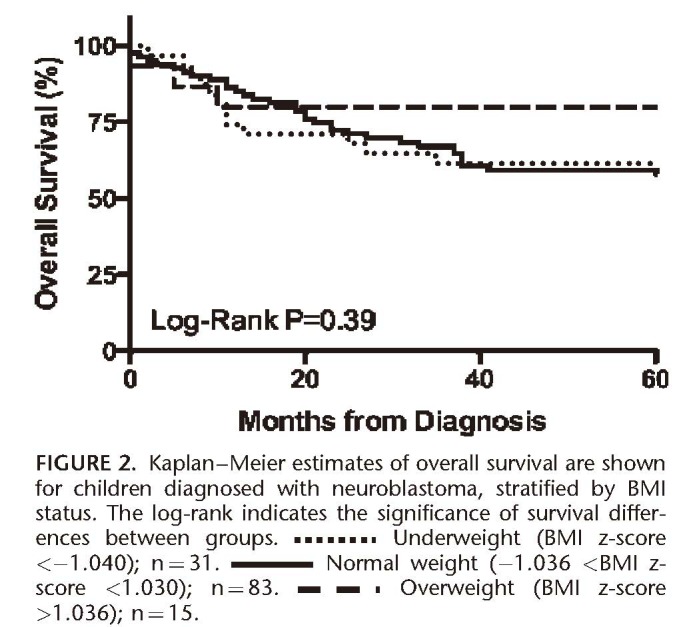


The Kaplan–Meier estimated survival for the 154 children was 60.5% and 61.2% for the 129 children with weight and length/height available at diagnosis, Table 3. The unadjusted log-rank analysis indicated that there was no statistically significant difference in overall survival between children who were underweight, normal weight, or overweight at diagnosis, Figure 2. Stage of NB at diagnosis was the only statistically significant predictor of survival; children diagnosed with stage 4 were less likely to survive compared to those diagnosed with stage 4s (HR [95%CI]: 7.02 [1.7–29.0], *P* = .007). In multivariable modeling, none of the following were significant predictors of survival: decade of diagnosis (1.16 [0.6–2.2], *P* = .618), age at diagnosis (1.05 [0.9–1.2], *P* = 0.489), BMI z-score at diagnosis (0.89 [0.7–1.1], *P* = .219), nor being overweight (BMI z-score >1.036) at diagnosis (0.53 [0.17–1.7], *P* = .296). Similar results were found if weight z-score replaced BMI z-score in the model, data not shown.

**TABLE 3:**
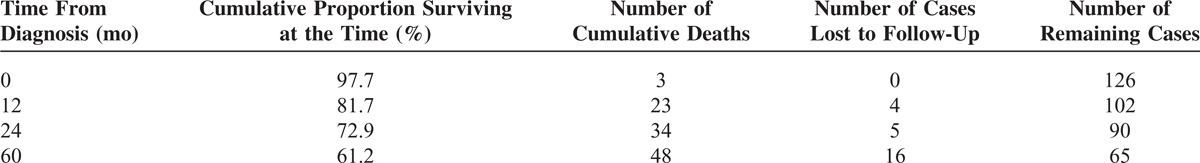
Kaplan–Meier Survival Estimates for 129 Children Diagnosed With Neuroblastoma With Recorded BMI at Diagnosis

## DISCUSSION

This study provides insight into the growth and BMI status of children diagnosed with NB. At diagnosis, almost a quarter (24%) of the children were categorized as underweight, suggesting a high incidence of malnutrition. The proportion of underweight children did not differ significantly by disease stage and is consistent with the reported late presentation of children with NB, with symptoms including anorexia and weight loss.^18^ However, the number of underweight children is less than that described in an earlier study from the 1980s which indicated malnutrition to be as high as 50% at diagnosis in children with stage 4.^19^ The disparity in the findings may indicate underlying differences in study populations or that children included in this review had an earlier diagnosis.

Six months after diagnosis all children, except those with stage 4s disease, showed a significant decrease in age and sex adjusted BMI. This outcome potentially reflects the difficulty in delivering sufficient calories to children with cancer, due to the metabolic effects of the malignancy combined with the side effects of multimodal therapy.^20^ Inadequate energy intake during childhood cancer treatment is associated with considerable short- and long-term effects. In addition to growth faltering, decreased tolerance to chemotherapy, higher susceptibility to infections and motor, cognitive and neurodevelopmental impairment have been reported.^20^ These results reinforce the need for intensive nutritional support during this period.

After 12 months, weight z-scores had begun to increase in children with all disease stages and increase in BMI z-scores was observed. This was particularly evident in children with stage 1 or 2 NB whose mean BMI increased by >1 z-score. Over the following 4 years BMI z-scores stabilized for children with stage 1 or 2 and 4s disease and gradually increased in children with stage 3 or 4 NB. The proportion of children who were classified as overweight doubled, from 12% at diagnosis to 28% at the last follow-up, approximately 5 years after diagnosis. High rates of obesity, which appear to be independent of age at diagnosis, gender, or treatment, have been well documented in survivors of acute lymphoblastic leukemia.^10^ The proportion of children who were classified as overweight in this study at follow up was also higher than that found in Australian children of a similar age, during a similar period of time, which indicate ∼20% were overweight.^21^ While a direct comparison is difficult to make due to population differences and different cut-points used to define BMI status, the results are concerning. Childhood cancer survivors tend to have an increased risk of a number of health conditions, including diabetes, metabolic disease, cardiovascular disease, and secondary cancers, all of which are associated with obesity and suggest further research into development of targeted interventions for obesity prevention is needed.^22–24^

At the last follow-up children with stage 1 or 2 and 4s NB had regained their stature, albeit relatively short (height z-score −0.52 [−1.1 to 0.2]) for children with stage 1 or 2 NB. Children with stage 3 or 4 disease, however, did not regain their relative height with a decrease in height of ∼0.8 z-score between diagnosis and last follow-up. This finding is consistent with results from smaller studies in children with advanced NB by Perwein et al^25^ (n = 31; median follow-up, 4.3 years) and Cohen et al^11^ (n = 51; median follow-up, 6.1 years), which showed significant decreases in height z-scores between diagnosis and last follow-up of approximately 0.3 and 0.8 to 1.9 (depending on treatment), respectively. Growth faltering in this population has been attributed to growth hormone insufficiency, hypothyroidism, intensive chemotherapy and irradiation, and/or inadequate nutrition.^11,25^

The second aim of this study was to determine if BMI at diagnosis of NB affected survival. In contrast to larger studies in children with other cancers,^7–9^ this study did not find any prognostic association between BMI and survival of children diagnosed with NB. A retrospective study of 768 pediatric acute myeloid leukemia patients found that children with both a low BMI (≤10th percentile) and high BMI (≥95th percentile) at diagnosis were less likely to survive, compared to children with BMI in the 11th to 94th percentiles.^8^ Another retrospective study of 4260 acute lymphoblastic leukemia patients found that BMI >95th percentile was associated with increased risk of relapse and decreased 5-year event-free survival in children older than 10 years at diagnosis.^7^ The mechanisms underlying the association between BMI and survival are not well understood, but are thought to be the result of altered drug toxicity, and variations in the pharmacokinetics of chemotherapeutic drugs according to adiposity.^20^ The lack of association found in this study may be due to the aggressive biological behavior of NB and/or differences in treatment protocols compared with other childhood cancers. During the study period, at the Sydney Children's Hospital (Westmead), treatment for NB varied by age and stage. Localized stage 1 and 2 tumors with favorable biological features were treated with surgery, and invasive locoregional stage 3 tumors were treated with chemotherapy and surgery. Infants (<12 months of age) with stage 4 disease received chemotherapy. Older children with stage 4 disease were managed with multimodality therapy including surgery, chemotherapy, radiation, and for those diagnosed after 1987 an autologous bone marrow transplant. Tumors with N-myc proto-oncogene protein amplification were also treated with multimodality therapy.

To our knowledge, this is the largest study of growth and BMI status in children with NB. The study's strengths include the relatively large cohort size and the comprehensive long-term follow-up and survival data. Length/height and weight were frequently recorded in the children's medical records, allowing the weight patterns and BMI status of the children to be compared during this time. This study also had some limitations. The small number of children with a BMI >95th percentile prevented a subanalysis of this group which has been used in the previous studies. Secondly, BMI is an imprecise measure of adiposity. Results from a recent study showed that BMI underestimated the incidence of malnutrition and obesity in children diagnosed with hematologic malignancies and solid tumors.^26^ The anthropometric measurements used may also have been compromised by difficulties obtaining accurate length measurements in infants, and by disease factors such as tumor size or changes in total body water caused by malignancy.^27,28^ The study was also limited by its retrospective design, as it relied on routinely collected data that limited the availability of information, such as parental heights and sociodemographic factors, which could have affected the children's growth outcomes. Future prospective studies using more accurate measures of adiposity such as dual x-ray absorptiometry or bioelectrical impedance would be beneficial to understanding the association between adiposity and prognosis in children diagnosed with NB.

In conclusion, a low BMI in children with NB is common at diagnosis and during treatment. However, we did not demonstrate a prognostic association between BMI status and survival. The high proportion of children diagnosed with NB who were overweight at long-term follow-up highlights the need for nutritional interventions to prevent late effects and optimize the health for these children.
